# Food insecurity and suicidal behaviours among Bangladeshi university students: a multi-institutional cross-sectional study

**DOI:** 10.1017/S1368980024001137

**Published:** 2024-05-23

**Authors:** Nitai Roy, Md. Aktarujjaman, Aysha Siddiky, Kakali Mollick, Sultan Mahmud Imran, Mohammed A. Mamun

**Affiliations:** 1 Department of Biochemistry and Food Analysis, Patuakhali Science and Technology University, Patuakhali, Bangladesh; 2 Faculty of Nutrition and Food Science, Patuakhali Science and Technology University, Patuakhali, Bangladesh; 3 CHINTA Research Bangladesh, Dhaka, Bangladesh; 4 Department of Public Health and Informatics, Jahangirnagar University, Dhaka, Bangladesh

**Keywords:** Food insecurity, Suicidal behaviours, University students, Bangladesh

## Abstract

**Objective:**

Suicidal behaviours among students pose a significant public health concern, with mental health problems being well-established risk factors. However, the association between food insecurity (FIS) and suicidal behaviours remains understudied, particularly in Bangladesh. This study aimed to investigate the relationship between FIS and suicidal behaviours among Bangladeshi university students.

**Design:**

A cross-sectional survey using convenience sampling was conducted between August 2022 and September 2022. Information related to socio-demographics, mental health problems, FIS and related events and suicidal behaviours were collected. Chi-squared tests and multivariable logistic regression models, both unadjusted and adjusted, were employed to examine the relationship between FIS and suicidal behaviour.

**Setting:**

Six public universities in Bangladesh.

**Participants:**

This study included 1480 students from diverse academic disciplines.

**Results:**

A substantial proportion of respondents experienced FIS, with 75·5 % reporting low or very low food security. Students experiencing FIS had a significantly higher prevalence of suicidal ideation, plans and attempts compared with food-secure students (18·6 % *v*. 2·8 %, 8·7 % *v*. 0·8 % and 5·4 % *v*. 0·3 %, respectively; all *P* < 0·001). In addition, students who have personal debt and participate in food assistance programmes had a higher risk of suicidal behaviours.

**Conclusions:**

This study highlights the association between FIS and suicidal behaviours among university students. Targeted mental health screening, evaluation and interventions within universities may be crucial for addressing the needs of high-risk students facing FIS.

## Introduction

Suicidal behaviours encompass ideation, plans and attempts, with ideation referring to thoughts of self-harm, plans indicating consideration of a specific suicide method and attempts involving engaging in potentially harmful actions with the intention of ending one’s own life^(1)^. Globally, approximately 800 000 individuals die by suicide each year, with the majority of these cases occurring in low- and middle-income countries, including Bangladesh^(2)^. Suicidal behaviour is prevalent among students, with a lifetime prevalence of ideation, plans and attempts reported at 22·3, 6·1 and 3·2 %, respectively, and 12-month prevalence rates at 10·6, 3·0 and 1·2 %^(3)^. Considering that suicide ranks as the fourth leading cause of death for individuals aged 15–29 years, which includes a significant proportion of university students, addressing suicidal behaviours in this population is of utmost importance^(2)^.

The most recent data on suicide rates in Bangladesh show alarming trends in suicide attempts and completions among youth. Furthermore, suicide rates have risen throughout the years, with those aged 18–30 years being the most vulnerable, particularly university students. The Bangladesh Bureau of Statistics estimated 11 000 suicide cases in 2021, showing a high suicide rate in Bangladesh^(4)^. The number of suicide attempts among university graduates in the country is increasing at an alarming rate. According to a report published by the Aachol Foundation in the Dhaka Tribune, at least 101 university students committed suicide in 2021, with male students accounting for 64·4 %^(5)^. Furthermore, 49 % of suicide deaths were among people aged 20–35 years. In 2022, about 532 suicide incidents were recorded, most of them were students^(6)^. In 2023, at least 513 students were from various educational institutions in the country committed suicide^(7)^. Out of the deceased, 227 (44·2 %) were school students, 140 (27·2 %) were college students, 98 (19·1 %) were university students, and 48 (9·4 %) were madrasa students. The report states that 60·2 % of the deceased were girls, while boys made up 39·8 % of the suicides recorded.

Various factors have been identified as associated with suicidal behaviours among Bangladeshi university students, including female gender, academic year, urban residence, substance use, mental disorders, Facebook addiction, physical and mental illness experiences, exposure to stressful life events, campus ragging, family mental illness history, hopelessness, perfectionism, family conflicts, relationship break-ups, lack of social support, financial crisis, comorbidity and family history of suicide^(8–11)^. Furthermore, the coronavirus disease 2019 pandemic and its related stressors have been linked to an increase in mental health problems and suicidal behaviour among university students, especially following the implementation of academic institution lockdowns^(12,13)^. Risk factors during the pandemic encompass aspects such as being female, experiencing sleep disturbances, smoking, having a family history of suicidal tendencies, having mental disorders, lower socio-economic status, urban living, physical inactivity, academic dissatisfaction, relationship complexities, emotional distress, conflict with family members, academic failure, mental health problems, sexual difficulties and parental scolding or restrictions^(12,13)^. However, despite the knowledge regarding numerous risk factors, food insecurity (FIS) among university students has received inadequate attention in the country.

University students face a higher risk of FIS compared with the general population, with prevalence rates ranging from 21 to 82 %^(14)^. Factors such as low-income backgrounds^(15)^, male gender^(16)^ and not living with parents^(17)^ disproportionately impact their vulnerability to FIS. Tuition increases, insufficient financial assistance and high living expenses contribute to FIS among students^(15)^, while factors such as financial trouble, cooking skills, poverty and unemployment have been mentioned in conceptual models related to FIS^(18)^. Student-specific risk factors, including higher housing and education costs, low income, inadequate financial resources, poor food management skills, increased reliance on borrowed funds and ineligibility for food assistance schemes, exacerbate FIS due to limited access to nutritious food required for health and academic performance^(19–21)^. Recent studies have revealed that students lack consistent access to affordable and nutritious food, leading to unhealthy eating practices and difficulty making healthy food choices^(16)^.

Experiences of FIS during young adulthood can have long-term consequences, including academic struggles, lower grades, poor concentration, course withdrawals or suspensions, compromised nutritional status, unhealthy dietary habits, lower self-reported health and increased risk of chronic illnesses^(14,22)^. FIS is also associated with an elevated risk of mental health issues^(14,23)^, higher mortality rates and increased suicidal behaviours among adults^(24,25)^. However, the existing knowledge regarding the relationship between FIS and suicidal behaviour among students primarily stems from studies conducted outside of Bangladesh^(26–28)^ or is extrapolated from research involving teenagers and older individuals^(25,29,30)^. Consequently, there is a critical research gap concerning the association between FIS and suicidality among students, which is essential to address given that suicide and accidental self-harm are the leading preventable causes of premature death among youths.

Suicide among students poses a significant public health threat, necessitating an assessment of the extent and scope of the problem experienced by students across various campuses in Bangladesh to inform targeted interventions. To date, no research has examined the connection between FIS and suicidal behaviours among Bangladeshi students. Therefore, this study represents a crucial initial step in understanding the adverse impacts of FIS on students’ well-being. Beyond the well-established psychological variables, the primary objective of this study is to evaluate the relationship between FIS and suicidal behaviours among university students, contributing to the understanding of this complex issue.

## Methods

### Study design, procedure and participants

This cross-sectional study utilised a convenience sampling method and included participants from six socio-economically and regionally diverse public universities in Bangladesh. Public universities were selected due to their typically larger and more diverse student population compared with private universities, which allows for a broader sample in the study. The participating universities were Patuakhali Science and Technology University, Bangabandhu Sheikh Mujibur Rahman Science and Technology University, Jahangirnagar University, Jagannath University, Barisal University and Rajshahi University. Each university offered a unique programme, focusing on agriculture, fisheries, science, technology, arts, sciences, social sciences and research and innovation in various fields. The study aimed to recruit undergraduate and master’s level students, with an average age of 21·73 years (sd ± 1·56 years), to assess FIS and its association with suicidal behaviours. Data collection took place between August 2022 and September 2022, with the classroom setting used for participant recruitment and data collection. The research team visited multiple classes to explain the study’s objectives and provide information on data collection and privacy. Students received explanatory materials and an informed consent form, ensuring the confidentiality of their information. However, self-reported data were collected through a pre-tested, validated questionnaire.

A total of 1600 students were selected using convenience sampling, representing the six participating universities. Out of the initial sample, 1505 individuals completed the survey. However, twenty-five incomplete responses were excluded, resulting in a final sample size of 1480. Inclusion criteria comprised students registered at the participating institutions, enrolled in traditional undergraduate and postgraduate programmes and representing diverse academic fields. This ensured a broad representation of academic subjects offered by the universities.

### Measures

#### Participant characteristics

Family income was categorised into three groups: ≤15 000 BDT (approximately $177), 15 000–30 000 BDT ($177–$354) and >30 000 BDT ($354). Participants were asked whether they receive any form of financial aid, such as scholarships, private or government loans or grants, to help cover tuition costs and related expenses, with response options of ‘yes’ or ‘no’. The study also gathered information on familial financial support, asking participants if their parents or other relatives provided them with financial assistance for university. Additionally, information on personal debt and financial dependence was collected. The participants’ grade point average (representing a student’s average performance across all their courses) was categorised as <3 or ≥3 (out of 4). The survey tool was distributed using self-administered surveys, allowing participants to provide responses independently through self-reporting methods.

#### Depression, anxiety and stress

This study used the Depression, Anxiety and Stress Scale (DASS-21) to assess depression, anxiety and stress levels^(31)^. The severity of symptoms was classified based on predetermined thresholds for mild, moderate-to-severe and severe symptoms. For depression, the cut-off points were normal (0–9), mild (10–13), moderate (14–20), severe (21–27) and extremely severe (+28). Similarly, for anxiety, the thresholds were normal (0–7), mild (8–9), moderate (10–14), severe (15–19) and extremely severe (+20). Stress symptoms were categorised as normal (0–14), mild (15–18), moderate (19–25), severe (26–33) and extremely severe (+34)^(32)^. The Cronbach’s alphas for the depression, anxiety and stress subscales in the Bangla-validated version were 0·99, 0·96 and 0·96, respectively^(32)^. In this study, the Cronbach’s α values were 0·93 for the overall DASS-21 scale, 0·85 for depression, 0·85 for anxiety and 0·86 for stress, indicating good reliability.

#### Food insecurity

To assess FIS, the United States Department of Agriculture (USDA) Adult Food Security Survey Module consisting of ten items was used^(33)^. The scale captures circumstances and behaviours related to anxiety about food supply, decreased food quality and quantity and meal skipping due to financial constraints. Participants’ responses were used to calculate a raw score ranging from 0 to 10, following the guidelines provided in the Guide to Measuring Food Security^(34)^. Based on the raw food security score, participants were categorised into four food security categories: high food security (raw score of 0, indicating no food access problems), marginal food security (raw score of 1 or 2, indicating anxiety over the food supply), low food security (raw score of 3–5, indicating reduced diet quality and variety) and very low food security (raw score of 6–10, indicating several indications of altered eating patterns and reduced food intake). For analysis purposes, the level of food security was dichotomised into two categories: food secure (comprising high and marginal food security) and food insecure (comprising low and very low food security). The Cronbach’s α value was 0·843 for the present study.

#### Suicidal behaviours

To assess suicidal behaviours, participants were asked a series of yes/no questions based on prior studies. They were asked about their experiences in the past 12 months, specifically whether they had seriously thought about trying to kill themselves (past-year suicidal ideation), made any plans to kill themselves (past-year suicidal plans) or attempted to kill themselves (past-year suicidal attempts)^(4)^. Positive responses were coded as ‘1’, indicating the presence of the respective suicidal behaviour, while negative responses were coded as ‘0’, indicating the absence of the behaviour. This approach is in line with well-established concepts of suicidality and reflects the assessment methodologies used in previous literature. This ensures that the evaluation of participants’ experiences with suicidal behaviours is consistent and comparable^(8,35)^. The Cronbach’s α of suicidal behaviour was 0·802 in the present study.

## Statistical analysis

For statistical analysis, the Statistical Package for Social Science (SPSS) version 28·0 was utilised. To begin, basic descriptive tests were performed to characterise the data (frequency, percentages and mean values with standard deviations). The χ2 (for all variables) test was used to examine the relationship between outcome variables and independent variables. Mann–Whitney U test were also applied to show the differences in the mean ranks of FIS with suicidal behaviors. Multicollinearity was checked, and all variables were incorporated into binary regression tests with past-year suicidal ideation, suicidal plans and suicide attempts as outcome variables. The Hosmer–Lemeshow tests (*P* ≥ 0·05) were used to assess model fitness (the *P*-values were 0·717, 0·873 and 0·421 for suicidal ideation, suicidal plan and suicidal attempt, respectively). In this study, tests were performed with 95 % CI, and *P*-values less than 0·05 were considered significant.

## Results

Table 1 presents the characteristics of the study sample. Participant characteristics such as age, gender, monthly family income, father’s education level and mother’s education level were collected using a semi-structured questionnaire. The study included 1480 university students with a mean age of 21·73 ± 1·56 years. The majority of participants were male (75·1 %), and 62·0 % were between the ages of 21 and 23 years. Regarding household income, 57·2 % of participants came from middle-class households (15 000–30 000 BDT). In terms of academic performance, 71·9 % of students maintained a grade point average of 3·0 or higher. Additionally, 72·0 % had no personal debt, 64·9 % received financial support from their families, 84·3 % did not receive financial aid, 82·6 % did not participate in food assistance programmes, and 65·3 % were not financially independent. In terms of food security, 24·5 % of students reported high/marginal food security, 37 % reported low food security, and 38·5 % reported very low food security.

**Table 1:** Baseline characteristics of the study population (*n* 1480)

Variables	Categories	Total	
		*n*	%
Gender	Male	1112	75·1
	Female	368	24·9
Age in years (mean = 21·73, sd = 1·56)	Below 21	374	25·3
	21–23	918	62·0
	Above 23	188	12·7
Monthly family income (BDT)	Below 15 000	424	28·6
	15 000–30 000	846	57·2
	Above 30 000	210	14·2
Father’s education	No formal education	169	11·4
	Primary	256	17·3
	Secondary	363	24·5
	Higher secondary	399	27·0
	Hons or above	293	19·8
Mother’s education	No formal education	215	14·5
	Primary	477	32·2
	Secondary	476	32·2
	Higher secondary	229	15·5
	Hons or above	83	5·6
Current CGPA	Below 3	413	27·9
	Equal or above 3	1067	72·1
Debt	No	1065	72·0
	Yes	415	28·0
Familial financial support	No	520	35·1
	Yes	960	64·9
Receive financial aid	No	1247	84·3
	Yes	233	15·7
Food assistance programmed participation	No	1222	82·6
	Yes	258	17·4
Financially independent	No	966	65·3
	Yes	514	34·7
Food security status	Food insecure	1117	75·5
	Food secure	363	24·5
Depression	No	662	44·7
	Yes	818	55·3
Anxiety	No	528	35·7
	Yes	952	64·3
Stress	No	1004	67·8
	Yes	476	32·2

*Note*: CGPA, Cumulative Grade Point Average.

Table 2 presents the findings derived from the Mann–Whitney *U* test. The results of the *U* test indicate a significant difference between FIS scores, in terms of suicidal ideation (*U* = 91·2, *P* < 0·001), suicidal plans (*U* = 56·6, *P* < 0·001) and suicidal attempts (*U* = 36·1, *P* < 0·001). The recorded scores consistently showed a pattern of being relatively higher among the affirmative groups across all cases.

**Table 2:** Results of the Mann–Whitney *U* test for the differences in the mean ranks of suicidal behaviours

Suicidal behaviours	Categories	Food insecurity mean	sd	Mean rank	*U* test value	*P*-value†
Past-year suicidal ideation	No	4·31	2·68	696·82	91·2	<0·001
	Yes	6·2	2·35	993·36		
Past-year suicidal plans	No	4·44	2·69	718·2	56·6	<0·001
	Yes	6·59	2·34	1048·21		
Past-year suicidal attempts	No	4·49	2·7	726·79	36·1	<0·001
	Yes	6·69	2·39	1059·34		

† *P*-value derived from Mann–Whitney *U* test.

Table 3 presents the bivariate association between past-year suicidal behaviours and independent variables. The prevalence of past-year suicidal ideation, plans and attempts were 14·7, 6·8 and 4·1 %, respectively. All types of suicidal behaviours were significantly more prevalent among university students experiencing FIS compared with those who were food secure (18·6 % *v*. 2·8 %; 8·7 % *v*. 0·8 %; and 5·4 % *v*. 0·3 %, respectively; *P* < 0·001). Furthermore, monthly income, father’s education, mother’s education, personal debt, participation in food assistance programmes, financial independence, depression and anxiety showed significant associations with suicidal ideation, plans and attempts (*P* < 0·001). In addition, current grade point average (*P* = 0·021), receiving financial aid (*P* = 0·003) and stress (*P* < 0·001) were found to be significantly associated with suicidal ideation only.

**Table 3:** Distribution of the variables with suicidal behaviours

Variables	Past-year suicidal ideation (*n* 218; 14·7 %)				Past-year suicidal plans (*n* 100; 6·8 %)				Past-year suicidal attempts (*n* 61; 4·1 %)			
	Yes, *n*	%	χ^2^ test value	*P*-value	Yes, *n*	%	χ^2^ test value	*P*-value	Yes, *n*	%	χ^2^ test value	*P*-value
Gender												
Male	160	14·4	0·42	0·520	77	6·9	0·20	0·655	49	4·4	0·92	0·338
Female	58	15·8			23	6·3			12	3·3		
Age in years												
Below 21	65	17·4	5·30	0·071	25	6·7	0·77	0·680	17	4·5	0·27	0·876
21–23	134	14·6			65	7·1			37	4·0		
Above 23	19	10·1			10	5·3			7	3·7		
Monthly family income (BDT)												
Below 15 000	76	17·9	24·21	<0·001	35	8·3	6·64	0·036	23	5·4	7·20	0·027
15 000–30 000	134	15·8			59	7·0			36	4·3		
Above 30 000	8	3·8			6	2·9			2	1·0		
Father’s education												
No formal education	52	30·8	52·77	<0·001	28	16·6	35·89	<0·001	19	11·2	30·56	<0·001
Primary	47	18·4			23	9·0			15	5·9		
Secondary	47	12·9			19	5·2			11	3·0		
Higher secondary	51	12·8			19	4·8			10	2·5		
Hons or above	21	7·2			11	3·8			6	2·0		
Mothers’ education												
No formal education	56	26·0	29·43	<0·001	30	14·0	24·47	<0·001	21	9·8	24·90	<0·001
Primary	72	15·1			34	7·1			22	4·6		
Secondary	58	12·2			19	4·0			9	1·9		
Higher secondary	24	10·5			13	5·7			7	3·1		
Hons or above	8	9·6			4	4·8			2	2·4		
Current CGPA												
Below 3	75	18·2	5·37	0·021	35	8·5	2·68	0·101	20	4·8	0·75	0·385
Equal or above 3	143	13·4			65	6·1			41	3·8		
Debt												
No	125	11·7	27·08	<0·001	56	5·3	13·54	<0·001	29	2·7	18·80	<0·001
Yes	93	22·4			44	10·6			32	7·7		
Familial financial support												
No	87	16·7	2·56	0·110	44	8·5	3·70	0·054	27	5·2	2·33	0·127
Yes	131	13·6			56	5·8			34	3·5		
Receive financial aid												
No	169	13·6	8·74	0·003	78	6·3	3·17	0·075	46	3·7	3·75	0·053
Yes	49	21·0			22	9·4			15	6·4		
Food assistance programmed participation												
No	161	13·2	13·49	<0·001	74	6·1	5·47	0·019	41	3·4	10·42	0·001
Yes	57	22·1			26	10·1			20	7·8		
Financially independent												
No	178	18·4	30·26	<0·001	80	8·3	10·27	0·001	48	5·0	5·05	0·025
Yes	40	7·8			20	3·9			13	2·5		
Food security status												
Food insecure	208	18·6	54·91	<0·001	97	8·7	26·85	<0·001	60	5·4	18·00	<0·001
Food secure	10	2·8			3	0·8			1	0·3		
Depression												
No	62	9·4	27·44	<0·001	26	3·9	15·22	<0·001	16	2·4	8·81	0·003
Yes	156	19·1			74	9·0			45	5·5		
Anxiety												
No	35	6·6	42·89	<0·001	14	2·7	21·96	<0·001	11	2·1	8·63	0·003
Yes	183	19·2			86	9·0			50	5·3		
Stress												
No	115	11·5	26·67	<0·001	59	5·9	3·84	0·050	40	4·0	0·15	0·699
Yes	103	21·6			41	8·6			21	4·4		
Total	218	14·7			100	6·8			61	4·1		

Note: *P*-values calculated using χ2 tests.

CGPA, Cumulative Grade Point Average.

Table 4 presents the adjusted model examining the influence of variables on suicidal behaviours. In the adjusted model for suicidal ideation, students who were food secure had a 75 % lower risk of suicidal ideation compared with those who were food insecure (AOR = 0·25, 95 % CI: 0·12, 0·54). Students with anxiety had a 1·95-times increased risk, while those with stress had a 1·42-times increased risk. Additionally, students whose fathers had no formal education had a 4·70-times higher risk, those with personal debt had a 1·49-times higher risk, those receiving financial aid had a 1·77-times higher risk, and those participating in food assistance programmes had a 1·87-times higher risk of suicidal ideation. Furthermore, students who were financially independent had a 43 % lower risk (AOR = 0·57, 95 % CI: 0·36, 0·9), males had a 34 % lower risk (AOR = 0·66, 95 % CI: 0·46, 0·94), and students whose fathers had no formal education had a 4·70-times lower risk of suicidal ideation.

**Table 4:** Logistic regression analysis of the variables associated with suicidal behaviours

Variables	Past-year suicidal ideation						Past-year suicidal plans						Past-year suicidal attempts					
	Unadjusted model			Adjusted model (Nagelkerke’s *R*^2^ = 0·193)			Unadjusted model			Adjusted model (Nagelkerke’s *R*^2^ = 0·148)			Unadjusted model			Adjusted model (Nagelkerke’s *R*^2^ = 0·157)		
	*P*-value	COR	95 % CI	*P*-value	AOR	95 % CI	*P*-value	COR	95 % CI	*P*-value	AOR	95 % CI	*P*-value	COR	95 % CI	*P*-value	AOR	95 % CI
Food security status																		
Food secure	<0·001	0·12	0·07, 0·24	<0·001	0·25	0·12, 0·54	<0·001	0·09	0·03, 0·28	0·001	0·11	0·03, 0·40	0·003	0·05	0·01, 0·35	0·024	0·08	0·01, 0·72
Food insecure		Reference			Reference			Reference			Reference			Reference			Reference	
Depression																		
Yes	<0·001	2·28	1·67, 3·12	0·077	1·41	0·96, 2·07	<0·001	2·43	1·54, 3·85	0·093	1·58	0·93, 2·71	0·004	2·35	1·32, 4·20	0·044	2·02	1·02, 4·01
No		Reference			Reference			Reference			Reference			Reference			Reference	
Anxiety																		
Yes	<0·001	3·35	2·30, 4·90	0·003	1·95	1·26, 3·02	<0·001	3·65	2·05, 6·48	0·009	2·38	1·24, 4·57	0·005	2·61	1·34, 5·05	0·278	1·53	0·71, 3·27
No		Reference			Reference			Reference			Reference			Reference			Reference	
Stress																		
Yes	<0·001	2·14	1·59, 2·86	0·043	1·42	1·01, 1·99	0·051	1·51	1·00, 2·29	0·628	0·89	0·56, 1·42	0·699	1·11	0·65, 1·91	0·208	0·68	0·38, 1·24
No		Reference			Reference			Reference			Reference			Reference			Reference	
Gender																		
Male	0·520	0·90	0·65, 1·25	0·023	0·66	0·46, 0·94	0·655	1·12	0·69, 1·81	0·629	0·88	0·53, 1·48	0·340	1·37	0·72, 2·60	0·853	1·07	0·54, 2·11
Female		Reference			Reference			Reference			Reference			Reference			Reference	
Age in years																		
Below 21	0·024 0·106	1·87	1·09, 3·23	0·140 0·226	1·55	0·87, 2·79	0·528 0·383	1·28	0·60, 2·71	0·924 0·618	1·04	0·47, 2·30	0·650 0·844	1·23	0·50, 3·02	0·989 0·965	0·99	0·38, 2·57
21–23		1·52	0·91, 2·53		1·40	0·81, 2·40		1·36	0·68, 2·69		1·20	0·59, 2·45		1·09	0·48, 2·47		0·98	0·41, 2·33
Above 23		Reference			Reference			Reference			Reference			Reference			Reference	
Monthly family income (BDT)																		
Below 15 000	<0·001 <0·001	5·51	2·61, 11·66	0·711 0·403	1·20	0·46, 3·09	0·013 0·032	3·06	1·27, 7·39	0·177 0·297	0·45	0·14, 1·44	0·016 0·036	5·97	1·39, 25·55	0·828 0·928	0·82	0·14, 4·73
15 000–30 000		4·75	2·29, 9·86		1·46	0·60, 3·55		2·55	1·09, 5·99		0·56	0·19, 1·66		4·62	1·10, 19·35		0·93	0·18, 4·89
Above 30 000		Reference			Reference			Reference			Reference			Reference			Reference	
Father’s education																		
No formal education	<0·001 <0·001 0·017 0·018	5·76	3·32, 9·99	0·001 0·025 0·078 0·095	4·70	1·83, 12·11	<0·001 0·014 0·369 0·521	5·09	2·46, 10·52	0·025 0·096 0·227 0·402	4·14	1·19, 14·42	<0·001 0·026 0·434 0·692	6·06	2·37, 15·49	0·164 0·301 0·513 0·674	3·16	0·63, 16·01
Primary		2·91	1·69, 5·02		2·75	1·14, 6·62		2·53	1·21, 5·30		2·77	0·83, 9·21		2·98	1·14, 7·79		2·29	0·48, 11·05
Secondary		1·93	1·12, 3·30		2·10	0·92, 4·77		1·42	0·66, 3·03		2·03	0·64, 6·42		1·50	0·55, 4·09		1·67	0·36, 7·68
Higher secondary		1·90	1·12, 3·23		1·85	0·90, 3·82		1·28	0·60, 2·74		1·53	0·57, 4·09		1·23	0·44, 3·42		1·32	0·36, 4·86
Hons or above		Reference			Reference			Reference			Reference			Reference			Reference	
Mother’s education																		
No formal education	0·003 0·194 0·508 0·829	3·30	1·50, 7·28	0·031 0·007 0·028 0·170	0·25	0·07, 0·88	0·034 0·443 0·726 0·768	3·20	1·09, 9·39	0·181 0·074 0·066 0·487	0·32	0·06, 1·70	0·049 0·369 0·754 0·763	4·38	1·01, 19·13	0·526 0·321 0·169 0·640	0·49	0·05, 4·46
Primary		1·67	0·77, 3·60		0·20	0·06, 0·64		1·52	0·52, 4·39		0·24	0·05, 1·15		1·96	0·45, 8·49		0·34	0·04,2·86
Secondary		1·30	0·60, 2·84		0·29	0·10, 0·87		0·82	0·27, 2·48		0·24	0·05, 1·10		0·78	0·17, 3·68		0·24	0·03, 1·83
Higher secondary		1·10	0·47, 2·55		0·49	0·18, 1·36		1·19	0·38, 3·75		0·62	0·17, 2·36		1·28	0·26, 6·27		0·65	0·11, 3·95
Hons or above		Reference			Reference			Reference			Reference			Reference			Reference	
Current CGPA																		
Below 3	0·021	1·43	1·06, 1·95	0·639	0·92	0·65, 1·30	0·103	1·43	0·93, 2·19	0·835	0·95	0·60, 1·52	0·386	1·27	0·74, 2·20	0·378	0·77	0·42, 1·39
Equal or above 3		Reference			Reference			Reference			Reference			Reference			Reference	
Debt																		
Yes	<0·001	2·17	1·61, 2·92	0·017	1·49	1·07, 2·07	<0·001	2·14	1·42, 3·23	0·120	1·43	0·91, 2·23	<0·001	2·99	1·78, 5·00	0·023	1·91	1·10, 3·34
No		Reference			Reference			Reference			Reference			Reference			Reference	
Familial financial support																		
Yes	0·110	0·79	0·59, 1·06	0·564	1·10	0·79, 1·54	0·056	0·67	0·45, 1·01	0·717	0·92	0·58, 1·45	0·130	0·67	0·40, 1·12	0·752	0·91	0·51, 1·62
No		Reference			Reference			Reference			Reference			Reference			Reference	
Receive financial aid																		
Yes	0·003	1·70	1·19, 2·42	0·008	1·77	1·16, 2·70	0·077	1·56	0·95, 2·56	0·181	1·49	0·83, 2·65	0·056	1·80	0·99, 3·28	0·395	1·37	0·66, 2·83
No																		
Food assistance programmed participation																		
Yes	<0·001	1·87	1·33, 2·62	0·002	1·87	1·26, 2·76	0·021	1·74	1·09, 2·78	0·048	1·71	1·01, 2·92	0·002	2·42	1·39, 4·21	0·009	2·34	1·24, 4·43
No		Reference			Reference			Reference			Reference			Reference			Reference	
Financially independent																		
Yes	<0·001	0·37	0·26, 0·54	0·016	0·57	0·36, 0·90	0·002	0·45	0·27, 0·74	0·430	0·78	0·42, 1·45	0·027	0·50	0·27, 0·93	0·758	0·89	0·41, 1·93
No		Reference			Reference			Reference			Reference			Reference			Reference	

Note: AOR, adjusted OR; CGPA, Cumulative Grade Point Average; COR, crude OR. Reference category is no.

In the adjusted model for suicidal plans, students who were food secure had an 89 % lower risk of planning suicide compared with those who were food insecure (AOR = 0·11, 95 % CI: 0·03, 0·40). Additionally, students with anxiety had a 2·38-times higher risk, those with uneducated fathers had a 4·14-times higher risk, and those participating in food assistance programmes had a 1·71-times higher risk of suicidal plans. Furthermore, in the adjusted model for past-year suicidal attempts, the food-secure group had a 92 % lower likelihood of attempting suicide compared with the food-insecure group (AOR = 0·08, 95 % CI: 0·01, 0·72). Furthermore, students who were depressed had a 2·02-times higher likelihood, those with personal debt had a 1·91-times higher likelihood, and those participating in food assistance programmes had a 2·34-times higher likelihood of attempting suicide (Table 4).

## Discussion

In this study, a substantial proportion of respondents experienced FIS, with 75·5 % reporting low or very low food security. This prevalence is higher than rates reported in previous studies conducted studies elsewhere. For instance, the prevalence ranges from 35 to 42 % among postsecondary students^(36)^ and 21–82 % among undergraduate and graduate students^(14)^, as estimated by recent systematic reviews. Similarly, a cross-national study^(26)^ have reported moderate (46·7 %) and severe (7·0 %) FIS among adolescents attending school. The increased prevalence seen in this study may be attributed to various contributing factors, such as socio-economic background, regional disparities or other contextual characteristics that could influence FIS among university students. Given the higher prevalence of FIS in this sample, it is important to investigate its impact on students’ well-being, particularly regarding the understudied relationship between FIS and suicidal behaviour. This study aims to fill this research gap and provide insights into the association between FIS and extreme mental health outcomes, suicidal behaviours, among university students, with the goal of identifying potential areas for intervention and support.

In our study, we observed a prevalence of 18·6 % for last-year suicidal ideation, 8·7 % for suicidal plans and 5·4 % for suicidal attempts among the university student population. These findings are comparable with the studies conducted in high-income countries such as the USA^(27)^, Taiwan^(37)^ and Canada^(38)^, as well as studies in low-income countries of Benin^(39)^, Lebanon^(33)^ and Tanzania^(28)^. Besides, this study’s findings support the existing evidence highlighting the association between FIS and suicidal behaviours among students. Studies utilising data from the Global School-based Student Health Survey found a connection between FIS and past-year suicidal behaviours^(30)^, such as ideation and plan^(28)^, and suicidal attempts^(26)^. Besides, the analysis of 2008 data from Wave IV of the National Longitudinal Study of Adolescent to Adult Health revealed a significant association between FIS and suicidal ideation among young adults aged 24–32 years^(40)^. In the USA, the 2017 Youth Risk Behavior Survey involving high school students from eleven states also reported a link between FIS, suicidal behaviour and mental health^(27)^. The consistent findings from this study and prior research highlight the importance of addressing FIS as a potential risk factor for suicidal behaviours among university students. It emphasises the need for comprehensive strategies and interventions that address both the psychological well-being and the nutritional needs of students. By implementing targeted programmes and support services aimed at reducing FIS and promoting mental health, universities can contribute to the prevention of suicidal behaviours and the overall well-being of their student populations.

There are a number of pathways that can help explain the connection between FIS and suicidal behaviours. First, inadequate nutrition has been associated with a higher risk of mental health issues, including suicidal behaviours. Limited access to food often leads to the consumption of cheaper, less nutritious options (e.g. higher in fats and carbohydrates, lower in vitamins and micronutrients, etc.)^(41)^, which can negatively impact mental well-being^(42)^. Second, the relationship between FIS and suicide may involve mental health factors such as depression, self-loathing, hopelessness and thoughts of suicide as a means of escape^(43)^. Depression can contribute to both poor dietary choices and suicidal tendencies^(44)^. FIS can lead to feelings of humiliation, anxiety and stress^(45)^, which can exacerbate mental disorders and increase the likelihood of suicidal behaviours. Besides, malnutrition and a lack of essential nutrients among food-insecure individuals may contribute to suicidal thoughts^(25)^. Further, the shame and stigma associated with experiencing material deprivation, such as a lack of food, may also play a role in increasing the risk of suicide^(46)^. While these psychological risk factors are well-established, further research is needed to fully understand the underlying mechanisms linking FIS between mental disorders and suicidal behaviours.

Participation in food assistance programmes has been associated with an increased risk of suicidal behaviour, although the reasons behind this link are not fully understood^(47)^. Individuals participating in US assistance programmes, such as the Supplemental Nutrition Assistance Programme, were found to have an increased risk of suicidal thoughts, planning and attempts^(47)^, as well as an independent association with greater depressive symptoms^(48)^. These associations remain significant even after controlling for various factors such as survey year, demographics, socio-economic status, health status and use of mental health services. This association may be due, in part, to the fact that students who are under financial stress and who rely on food assistance are more likely to suffer from thoughts of suicide due to the accompanying emotions of guilt, shame, helplessness and exposure to unwanted contact with other recipients. Further studies into the characteristics of food assistance programmes that mitigate the risk of embarrassment could be a promising direction for the future.

Regarding student debt, studies have produced findings regarding its association with psychological distress. For instance, a systematic review revealed that higher levels of debt were associated with depression, suicide, drug and alcohol addiction, as well as psychotic disorders^(49)^. In the present study, participants who reported having debt were more prone to experiencing suicidal behaviour, although the significance was not observed for suicidal plans in the adjusted model. The rise in student debt probably exacerbated frustration among students who could not satisfy expectations aligned with their personal goals. After earning a college degree, many students aim to secure a job that offers financial freedom to achieve life milestones like buying a house or car, establishing a family or saving for retirement. Therefore, debt might reduce the likelihood of graduates achieving their goals, potentially increasing the risk of suicide^(50)^. Situations can become even more problematic when students accumulate debt without completing their degrees, which can worsen repayment issues. Dealing with debt repayment can cause significant emotional distress, including feelings of being overwhelmed, anxious, depressed and even thoughts of suicide among students. Nonetheless, further research is needed to provide a more comprehensive understanding of the relationship between student debt and suicidal behaviours.

This study has several limitations that should be acknowledged. First, the cross-sectional design of the study restricts us from establishing a causal relationship between FIS and suicidal behaviours. Prospective cohort studies would be necessary to understand how these processes unfold over time. Another limitation is the potential for recall and social desirability biases since participants were asked to recall their experiences of FIS and suicidal behaviours over the preceding 12 months. The reliance on self-reported data for both predictor and outcome variables introduces the possibility of response bias. Additionally, the sample used in this study consisted of self-selected participants, which may limit the generalisability of the findings to other college and university students in different contexts. The self-selected nature of the sample may affect the prevalence and severity of factors like FIS and suicidal behaviours, potentially leading to an overestimation of the association between FIS and suicidal behaviours. Our study has solely focused on public universities and has not included private ones. To enhance the robustness and applicability of future research, more diverse and representative samples should be employed, incorporating various demographic, cultural and institutional factors. This will provide a more comprehensive understanding of the relationship between FIS and suicidal behaviours across different student populations.

### Implications for research

This study highlights the importance of conducting additional research to enhance our comprehension of the intricate relationship between FIS and suicidal behaviours among university students, specifically in the context of Bangladesh. Longitudinal studies might play a crucial role in understanding the time-dependent nature of this relationship, shedding light on possible cause-and-effect mechanisms and pinpointing important factors that contribute to risk or provide protection^(22,40)^. Investigating the underlying factors that connect FIS to suicidal behaviours, including nutritional deficiencies, psychological distress and coping strategies, can offer valuable insights for focused interventions. Furthermore, a more thorough analysis of the correlation between debt and mental health outcomes, including various forms of debt and financial burdens, could provide valuable and nuanced insights^(49)^. Exploring various socio-economic and cultural contexts can provide valuable insights and a deeper understanding of the factors that influence this association. It would be beneficial for future research to assess the efficacy of current food assistance programmes in university settings and investigate the potential factors that contribute to a higher likelihood of suicidal behaviours among programme participants^(47)^.

### Implications for practice

The findings of this study have important implications for practical interventions within university settings. It is crucial to implement focused mental health screening programmes that cater to the unique requirements of students facing FIS. Universities must combine mental health support services with existing programmes that tackle FIS^(27)^. This will result in a comprehensive and holistic approach to promoting student well-being. Creating a supportive atmosphere on campus is crucial to addressing mental health concerns and FIS, ensuring that students are encouraged to seek assistance without any hesitation. Financial literacy programmes are valuable tools that can empower students to effectively manage debt and navigate financial stress. In addition, institutions should reassess and customise current food assistance programmes to not only meet nutritional needs but also integrate mental health resources and counselling services^(47)^. Collaboration between academic institutions and policymakers is essential for the development and implementation of policies aimed at addressing the socio-economic factors that contribute to FIS among students, thereby mitigating the associated risk of suicidal behaviours. Ultimately, universities should place high importance on fostering a nurturing and all-encompassing atmosphere that acknowledges and tackles the interrelated issues of mental health and FIS faced by their students^(27)^.

### Conclusion

In conclusion, this study highlights the significant prevalence of FIS among university students and its profound impact on their suicidal behaviours. The findings establish a strong relationship between FIS and related events with suicidal behaviours. This is particularly alarming considering the elevated vulnerability of university students to mental health issues due to the unique pressures of academic life. The implications of this research are crucial for policymakers and stakeholders to develop targeted interventions and policies addressing FIS both within households and educational settings. This may involve initiatives such as increasing government food funding, providing counselling and support services for food-insecure students and addressing the underlying socio-economic factors contributing to FIS. Moreover, institution-based suicide prevention programmes that promote social-emotional learning, foster social connections and offer parental support should be considered as essential components of comprehensive support for all students.

## Data Availability

The data supporting this study’s findings are available from the corresponding author upon reasonable request.
